# Risk factors of anemia among preschool children in Ethiopia: a Bayesian geo-statistical model

**DOI:** 10.1186/s40795-021-00495-3

**Published:** 2022-01-07

**Authors:** Bilal Shikur Endris, Geert-Jan Dinant, Seifu H. Gebreyesus, Mark Spigt

**Affiliations:** 1 School of Public Health, Department of Nutrition and Dietetics, Addis Ababa University, Addis Ababa, Ethiopia; 2 School CAPHRI, Care and Public Health Research Institute, Maastricht University, Maastricht, the Netherlands; 3 General Practice Research Unit, Department of Community Medicine, The Arctic University of Tromsø, Tromsø, Norway

## Abstract

**Background:**

The etiology and risk factors of anemia are multifactorial and varies across context. Due to the geospatial clustering of anemia, identifying risk factors for anemia should account for the geographic variability. Failure to adjust for spatial dependence whilst identifying risk factors of anemia could give spurious association. We aimed to identify risk factors of anemia using a Bayesian geo-statistical model.

**Methods:**

We analyzed the Ethiopian Demographic and Health Survey (EDHS) 2016 data. The sample was selected using a stratified, two- stage cluster sampling design. In this survey, 9268 children had undergone anemia testing. Hemoglobin level was measured using a HemoCue photometer and the results were recorded onsite. Based on the World Health Organization’s cut-off points, a child was considered anaemic if their altitude adjusted haemoglobin (Hb) level was less than 11 g/dL. Risk factors for anemia were identified using a Bayesian geo-statistical model, which accounted for spatial dependency structure in the data. Posterior means and 95% credible interval (BCI) were used to report our findings. We used a statistically significant level at 0.05.

**Result:**

The 9267 children in our study were between 6 and 59 months old. Fifty two percent (52%) of children were males. Thirteen percent (13%) of children were from the highest wealth quintile whereas 23% from the lowest wealth quintile. Most of them lived in rural areas (90%). The overall prevalence of anemia among preschool children was 57% (95% CI: 54.4–59.4). We found that child stunting (OR = 1.26, 95% BCI (1.14–1.39), wasting (OR = 1.35, 95% BCI (1.15–1.57), maternal anemia (OR = 1.61, 95% BCI (1.44–1.79), mothers having two under five children (OR = 1.2, 95% BCI (1.08–1.33) were risk factors associated with anemia among preschool children. Children from wealthy households had lower risk of anemia (AOR = 0.73, 95% BCI (0.62–0.85).

**Conclusion:**

Using the Bayesian geospatial statistical modeling, we were able to account for spatial dependent structure in the data, which minimize spurious association. Childhood Malnutrition, maternal anemia, increased fertility, and poor wealth status were risk factors of anemia among preschool children in Ethiopia. The existing anaemia control programs such as IFA supplementation during pregnancy should be strengthened to halt intergenerational effect of anaemia. Furthermore, routine childhood anaemia screening and intervention program should be part of the Primary health care in Ethiopia.

## Background

Globally, around two billion people are affected by anemia. Most of the anemia burden (more than 89%) occurs in developing countries primarily affecting children and women [1]. About half of preschool aged children and pregnant women are anemic. Moreover, more than half of the world’s population of preschool aged children and pregnant women live in countries where anemia is a severe public health problem, Africa and Asia being the most affected [2]. In Ethiopia, 57% of children aged 6–59 months are anemic, indicating that anemia is a severe public health problem [3].

The etiology of anemia is multifactorial, studies revealed iron deficiency anemia accounts for more than half of anemia burden globally [1]. Although nutritional factors are considered to be the key contributor to childhood anemia, their exact contribution to the risk of anemia is not well established and may vary with the level of infection and the diet quality. It has been estimated that about 37% of anemia cases in preschool children in three West African countries could be averted by treating nutrition related factors alone [4]. The proportion of anemia associated with iron deficiency was lower in countries where anemia is a severe public health problem, and in countries with very high inflammation [5]. A national micronutrient survey in Ethiopia reported that iron deficiency anemia (IDA) accounts for 35% of anemia cases (using sTfR (soluble serum transferrin receptor) [6]. A systematic review on risk factors of anaemia among children in Ethiopia revealed that low parental literacy; low socioeconomic, rural residence, and helminthic infection of the children were found to be predictors of anemia among children [7].

Infections are the other major cause of anemia after iron deficiency and may contribute up to 50% of the cases in some settings [8, 9]. Infections such as malaria and hookworms are highly prevalent in developing countries [10]. Studies conducted in many regions of Africa found positive associations between the presence and density of infection and anemia [11–13]. Studies showed Intestinal Parasites such as Hookworm, Ascaris lumbricoides, Trichuris trichiura are associated with anemia [14].. Studies carried out in different part of Africa among preschool children showed hookworm increased the risk of anemia [11, 12]. In West Africa, a risk mapping approach using geostatistical models estimated that 4.2% of anemia cases in preschool children could be averted by treating hookworm [15]. Accumulating evidence In Ethiopia showed intestinal Helminthes such as hookworm as a cause of anemia [16–19]. In the contrary to this, some studies showed no association between intestinal Helminthes and anemia [20–22] in Ethiopia. The lack of association in these studies could be explained by the low prevalence of parasite, low intensity of parasite and harboring of less invasive types of parasites. Malaria has also been found to be an important cause of anemia in areas with high malaria burden in Ethiopia [23–26]. However, malaria is less important cause of anemia in low malaria transmission setting of Ethiopia [16, 22, 27].

There are a number of individual, maternal, household and environmental risk factors of anemia among children. Accumulating evidence indicated that children from poorest household were at higher risk of anemia [28–31]. Many studies revealed that malnutrition is associated with anemia among preschool children [30, 32–37]. Malnutrition resulting from inadequate dietary intake and/or from infection and disease may also lead to anemia. Several hypotheses such as adaptation to lower tissue-metabolic requirements for oxygen transport, have been put forward to explain the relationship between anemia and protein-energy malnutrition (PEM) [38]. Some authors however consider that the anemia of PEM is the outcome of a complex hematological process in which iron and other micronutrient deficiencies interplay [39]. Maternal education is inversely associated with the prevalence of anemia [30, 40–42]. Some studies revealed that maternal anemia is a risk factor for anemia among children [35, 40].

Geographical variation in the etiology of anaemia can be explained in part by variation in environmental drivers of anaemia. Environmental factors that cause anaemia have a high degree of geographical clustering [5, 6]. For example, altitude and temperature are linked to the risk of malaria (a well-known cause of anaemia. Furthermore, the distance to a perennial water body, land surface temperature, and the normalized difference vegetation index (NDVI) are all linked to dietary iron deficiency and anaemia-causing helminthic infections [7].

Many studies indicate that anemia cases cluster geographically [43–47]. Geostatistical models are suitable for analyzing spatially correlated data such as anemia, as they take account into account spatial correlation by introducing location-specific random effects. The typical statistical models assume independence of observations. However, when spatially correlated data are analyzed this independence assumption, overestimates the statistical significance of the covariates [48]. On the other hand, the bayesian approach is valuable because the effect of covariates and spatial clustering can be modelled simultaneously and the uncertainty of predictions can be assessed. Furthermore, In addition to the incorporation of prior information and the ease in computation of complex models, Bayesian approach uses posterior probability [49]. Hence, the use of Bayesian Geo-statistical model is a preferred to come up with robust estimates of spatially correlated data.

Geospatial studies revealed geographical inequality of anemia in Ethiopia. Anemia hotspots were concentrated in the eastern part of the country including Somali, Dire dawa, Harari, and Afar regions [46, 47, 50]. Targeted approaches based on the geographical vulnerability and contextual risk factors are needed to efficiently allocate interventions and attain an accelerated reduction of anaemia in Ethiopia. In Ethiopia, although many studies were conducted to understand the risk factors of anemia, they did not account for the spatial dependence structures of anemia when analyzing risk factors. Failure to account for the spatial dependency could result in spurious association. Therefore, in the present work we aimed to identifying risk factors of anemia among children by accounting for the existing spatial dependency of the distribution of anemia among preschool children.

## Methods

### Study design and setting

We performed analysis of the Ethiopian Demographic and Health Survey (EDHS) 2016 data. The EDHS is carried out every 5 years to provide health and health-related indicators at the national and regional levels in Ethiopia. The 2016 EDHS sample was selected using a stratified, two- stage cluster sampling design. In the first stage, 645 clusters of census enumeration areas (EAs), including 202 urban areas, 443 rural areas were selected. In the second stage, 18,008 households were selected. In this survey, 9268 children had undergone anemia testing. The detailed sampling procedure is presented in the full EDHS report [51]. The EDHS 2016 data were downloaded from the DHS website (http://dhsprogram.com) after we secured online permission. In addition to the individual and household level data from the DHS, we used ecologic level data extracted from openly available DHS Program Spatial Data Repository (http://spatialdata.dhsprogram.com).

### Measurements

#### Outcome variable – child anemia

Anemia testing was performed from children 6 to 59 months from whom consent had been obtained from parents or another responsible guardian. For children 12 to 59 months of age, a drop of blood taken from the palm side of the end of a finger and for children age 6–11 months’ blood was taken from the heel prick. The blood samples were collected on HemoCue photometer and and the results were recorded onsite. In the current study, the outcome variable anemia is recoded into a dichotomous variable namely; having anemia: yes = 1 or not having anemia: no = 0 Based on the World Health Organization’s cut-off points, a child was considered anaemic if their altitude adjusted Hb level was less than 11 g/dL [51].

Explanatory variables.

We used both individual level and ecologic level data. Individual variables such as 1. Child variables: Age, sex, child disease (fever, diarrhea), dairy consumption, consumption of vegetables and fruits, and consumption of flesh food (liver, beef, poultry) of the child in the last 24 h before the survey, stunting and wasting, 2. Maternal variables: age, anemia, education, BMI, number of ANC visits, iron consumption during pregnancy, number of births, 3. household characteristics (improved water source, improved latrine, wealth status, residence). Ecologic level variables such as rainfall, temperature, enhanced vegetation index and altitude were included.

### Data analysis

The statistical analysis of the data has been performed using STATA 14. We applied Weight and complex survey design methods (SVY) as per DHS recommendation. Descriptive statistics were used to analyze baseline characteristics of children and their caregivers including sex, age, residence, mothers and partners education level and wealth index to provide an overall picture of the sample. The prevalence of each risk factor, and 95% confidence interval was also presented.

We ran bivariate analyses to select variables for the final model. Variables with *p*-value of less than 0.2 such as stunting, wasting, maternal anemia, maternal education, fertility (number of births less than 5 years old), household wealth status, availability of improved toilet, and amount of rainfall were used in the final bayesian geo-statistical model.

### Bayesian modeling

A Bayesian geostatistical model uses Markov chain Monte Carlo (MCMC) methods to avoid asymptotic Inference and the computational problems faced in likelihood-based fitting. The Bayesian approach allows parameter estimation using information coming from the data as well as information coming from other sources such as from previous studies or non-informative prior. It provides direct probability statements—which is not the case in the conventional statistics. In addition, Bayesian approach uses posterior probability. We can obtain estimates of the posterior distributions of both the regression coefficients (β) and function of the regression coefficients such as odds ratio. Posterior means and 95% credible intervals are used to summarize these posteriors [49, 52]. A spatial weight matrix conceptualizing the spatial relationship between clusters was generated using an inverse distance approach.

Bayesian Geo-statistical model was undertaken using WinBUGS version 1.4.3 (MRC Bio- statistics Unit, Cambridge and Imperial College London, UK). We started with non-informative prior and 10,000 iterations. We checked for convergence of parameters visually using history plot and kerenel density estimate. Convergence was successfully achieved after 10,000 iterations. A further 10,000 iterations were run and values were thinned by 10 and stored. The stored samples were used to calculate summary statistics (mean, SD, and 95% credible intervals) of the parameters. We used a statistically significant level at 0.05.

## Result

Table 1 shows the socio demographic characteristics of study participants. A total of 9267 children aged 6–59 months participated in this study. Fifty two percent (52%) of children were males and 48% were females. Thirteen percent (13%) of children were from the highest wealth quintile whereas to 23% from the lowest wealth quintile. Most of them lived in rural areas (90%). Regarding the characteristic of the mothers; 67% of mothers had no formal education.

**Table 1:** Socio demographic characteristics of study participants, Ethiopia

Socio-demographic Characteristics	Number	Percent
Child age in months		
6–11	1043	11.3
12–23	2022	21.8
24–35	1948	21.0
36–47	2019	21.8
48–59	2235	24.1
Sex		
Female	4455	48.0
Male	4812	52.0
Wealth quintile		
Poorest	2164	23.3
Poor	2166	23.4
Middle	1963	21.2
Rich	1723	18.6
Richest	1250	13.5
Mothers’ education		
No education	5746	67.1
Primary education	2307	26.9
Secondary education	345	4.0
Higher Education	170	2.0
Place of Residence		
Rural	8330	90
Urban	937	10
Total	9267	100

Table 2 shows prevalence of anemia across different children and maternal factors. Overall, the prevalence of anemia among preschool children was 57% (95%CI: 54.4–59.4). A higher prevalence of anemia was found among younger children (77% among 6–11 month vs. 40% among 48–59 month), Somali region (83% in Somali vs. 42% in Amhara), rural area (58% rural vs. 49% urban), lowest wealth quintile (68% poorest vs. 48% richest household), wasted children (66% wasted vs. 56% non-wasted).

**Table 2:** Prevalence of anemia among preschool children across different factors, Ethiopia

Factors	Prevalence of anemia with 95% confidence interval
Age in months	
6–11	77.1 (72.5–81.2)
12–23	69.2 (65.7–72.5)
24–35	59.0 (54.5–63.2)
36–47	50.9 (46.9–54.9)
48–59	40.0 (36.2–43.9)
Sex	
Female	56.6 (53.8–59.3)
Male	57.2 (54.1–60.3)
Region	
Tigray	53.6 (49.0–58.1)
Afar	74.8 (70.4–78.6)
Amhara	42.2 (37.9–46.5)
Oromiya	65.5 (61.0–69.6)
Somali	82.9 (79.6–85.8)
Benishangul-Gumuz	42.5 (37.6–47.6)
SNNPR	50.0 (45.0–54.9)
Gambela	56.2 (47.8–64.2)
Harari	67.9 (63.1–72.3)
Addis Ababa	49.2 (43.4–55.0)
Dire Dawa	71.5 (66.0–76.5)
Wealth quintile	
Poorest	67.8 (62.9–72.2)
Poor	57.6 (53.5–61.7)
Middle	52.6 (48.3–56.8)
Rich	54.0 (49.9–58.0)
Richest	47.9 (43.6–52.3)
Mothers’ education	
No education	58.5 (55.5–61.5)
Primary education	56.8 (53.3–60.3)
Secondary education	48.6 (41.7–55.6)
Higher Education	51.5 (40.2–58.1)
Place of Residence	
Rural	57.8 (55.1–60.5)
Urban	49.3 (43.5–53.1)
Stunting	
Severely stunted	65.3 (60.9–69.4)
Stunted	57.0 (52.8–61.1)
Not stunted	53.7 (51.1–56.3)
Wasting	
Severely wasted	72.0 (61.4–80.6)
Wasted	66.0 (59.4–72.0)
Not wasted	55.6 (53.3–58.2)
Maternal BMI	
< 18.5	60.5 (56.7–64.1)
18.5–24.9	56.6 (53.7–59.4)
25–29.9	53.7 (46.9–60.3)
> =30	53.8 (47.2–60.2)
Maternal anemia	
Anemic	68.9 (65.7–71.8)
Not anemic	52.3 (49.6–55.0)
Flesh food (Meat, poultry liver)	
No	63.6 (60.9–66.2)
Yes	62.4 (55.1–69.1)
Child ate Milk or dairy	
No	62.1 (59.1–65.0)
Yes	67.0 (62.8–70.7)
Child ate vegetables or fruits	
No	64.2 (61.1–67.1)
Yes	61.0 (55.9–65.8)
Child morbidity	
No	55.7 (52.8–58.5)
Yes	60.1 (60–63.2)
Improved water source	
No	57.8 (53.5–58.9)
Yes	56.3 (53.8–61.7)
Latrine	
No	57.1 (54.5–59.8)
Yes	55.2 (50.4–59.9)
Four or more Antenatal care	
No	60.6 (57.2–63.9)
Yes	58.9 (55.4–62.4)
Mother Received iron during pregnancy	
No	62.4 (58.6–66.0)
Yes	56.8 (53.8–59.8)
Number of births in the last 5 years	
1	52.9 (50.0–55.7)
2	60.8 (57.8–63.8)
3	61.0 (54.6–67.1)
4	70.1 (63.7–75.7)

The prevalence of anemia was higher among children of anemic mothers (69% for anemic mothers vs. 52% non anemic mothers), children of mothers with more than one under-five children (61% for two births vs. 53% for one birth).

### Risk factors of anemia among preschool children in Ethiopia

Table 3 displays the adjusted posterior odds ratio estimates (AOR) with their 95% credible intervals for the geo-additive model for anaemia. We found that children malnutrition, maternal and household economic status were significantly associated with anemia among preschool children.

**Table 3:** Risk factors of anemia among children in the in Ethiopia; a binary logistic geostatistical model

Bayesian logistic regression model with geo-statistical component				
Background characteristics	Posterior Mean	SD	Median	Adjusted 95% Bayesian credible intervals (BCI) OR (95% BCI)
***Number of children***				
1	*–*	*–*		1.0
2	1.20	0.05	1.20	1.20 (1.08–1.33)^*^
3	1.07	0.09	1.07	1.07 (0.90–1.27)
4	1.47	0.26	1.47	1.47 (0.90–2.44)
**Availability of improved toilet**				
No	–	–	–	1.0
Yes	1.05	0.08	1.05	1.05 (0.91–1.23)
**Maternal education**				
No education	–	–	–	1.0
Primary Education	1.05	0.06	1.05	1.05 (0.94,1.19)
Secondary Education	0.96	0.11	0.96	0.96 (0.78,1.18)
Higher education	0.85	0.15	0.85	0.85 (0.64,1.12)
**Child stunting**				
Not stunted	–	–	–	1.0
Stunted	1.26	0.05	1.26	1.26 (1.14,1.39)^*^
**Child wasting**				
Not wasted	–	–	–	1.0
Wasted	1.35	0.08	1.35	1.35 (1.15,1.57)^*^
**Maternal anemia**				
No anemia	–	–	–	1.0
Anemic	1.61	0.06	1.61	1.61 (1.44,1.79)^*^
**Household wealth**				
Poorest	–	–	–	1.0
Poor	0.81	0.07	0.81	0.81 (0.71–0.93)^*^
Middle	0.65	0.07	0.65	0.65 (0.56–0.75)^*^
Rich	0.73	0.08	0.73	0.73 (0.62–0.85)^*^
Richest	0.60	0.09	0.60	0.60 (0.50–0.71)
**Rainfall**				
Rainfall (mm)	0.99	0.0	0.99	0.99 (0.99,0.99)

* Significant association at *p* < 0.05

Childhood malnutrition such as stunting (OR = 1.26, 95% BCI (1.14–1.39) and wasting (OR = 1.35, 95% BCI (1.15–1.57) were risk factors of anemia. Stunted children had 1.3 times higher odds of anemia compared to non-stunted children. Similarly, wasted children had 1.4 times higher odds of anemia compared to non-wasted children.

Maternal factors such as maternal anemia (OR = 1.61, 95% BCI (1.44–1.79) and increased fertility (OR = 1.61, 95% BCI (1.44–1.79) were also found to be risk factors of anemia. On the other hand, children from wealthy household had lower risk of anemia (AOR = 0.73, 95% BCI (0.62–0.85).

## Discussion

This study aimed at identifying risk factors of anemia among preschool children using Bayesian geo-statistical modeling. We found that child malnutrition, maternal anemia, having more children, lower household wealth status were risk factors associated with anemia among preschool children.

Our previous work clearly demonstrated the spatial autocorrelation of anemia in Ethiopia [50]. We found considerable geographical variation; hotspots (clusters) of anemia were concentrated in the eastern part of Ethiopia. In the current study, we identified risk factors of anemia among children accounting for the existing spatial dependency of the distribution of anemia among preschool children. The use of Bayesian model in our study has multiple advantages over the conventional approach. The primary benefit of this model is its use of Markov chain Monte Carlo (MCMC) algorithms for Bayesian computation. Based on a large number of iteratively generated samples from MCMC, we obtained estimates of the posterior distribution of parameter of interest. Such estimates have more clear interpretation as the interval contain the true parameter with some probability. Another most important advantage of the Bayesian MCMC approach in our study is its flexibility to fit realistic models to our complex data of spatially correlated outcome (anemia) [49]. In the Bayesian model, we were able to account for spatial dependence structure of our data. Accounting for spatial correlation in the data helps to prevent spurious association between covariates and anemia [53].

Our finding of higher odds of anemia among malnourished children (stunted and wasted) is in consistent with other studies [30, 32–35, 54–56]. In a meta-analysis, a two times higher odds of anemia was found among stunted) and wasted children [54]. Several mechanisms can explain the higher odds of anemia among malnourished children. First, malnourished children have reduced metabolism as an adaptive response to the low dietary intake. The reduced metabolism results in low demand of oxygen and decreased production of red blood cells resulting in adaptive anemia [57]. The second possible reason is the inadequate dietary intake of nutrients that are essential for growth and hemoglobin synthesis can predispose children to be concurrently affected by malnutrition and anemia. Calorie deficient children are more likely to be deficient in other micronutrients including iron, the most important micronutrient for hemoglobin synthesis. In developing countries, in particular, where a diverse supply of foods is limited, both macro and micronutrients deficiencies are the common public health problems [57]. The third possible mechanism is related with impaired immunity of malnourished children which predispose them to infection. The infection in turn causes loss of nutrients, malabsorption, underutilization of bioavailable nutrients such as iron, blood loss and immune-mediated destruction of RBCs, which have been associated with anemia [54]. The fourth reason is related with similarity of risk factors between malnutrition and anemia such as poverty, poor sanitation and hygiene, poor health service, food insecurity, caretakers education, infection and dietary inadequacy, which predispose children for he concomitant burden of malnutrition and anemia [55]. The finding of higher odds of anemia among malnourished children implies that there is a need to integrate screening and intervention programs targeting malnutrition and anemia.

We found that anemic mothers have increased odds of having anemic child. Many other studies also reported similar finding that revealed the positive association between maternal and child anemia [35, 40, 58, 59]. Mothers and their children share similar socioeconomic, cultural and health related environment that can potentially expose them to be affected by anemia [60, 61]. For example, mothers might not afford to purchase good nutritious food for themselves as well as their children, which might result in anemia due to inadequate intake of iron, and other micronutrients [57]. On the other hand, both mothers and children could be exposed to helminthes, malaria and other infectious diseases that may interfere with their red blood cells production and iron stores [61]. In addition, there is high chance of intergenerational cycle of anemia from the mother to the infant. For example, maternal anemia during pregnancy contributes to low birth weight, which increases the risk of childhood anemia [62]. A study showed that the effect of maternal anemia on childhood anemia can extend even beyond 6 months age [63]. Furthermore, low levels of essential minerals such as iron in the breast milk of anemic mother could affect the Hb level of the breastfeeding child [64, 65]. Our finding of higher odds of anemia with increased parity could also be explained by cumulative fertility burden with unmet need of iron. Other studies also reported similar findings [66, 67] .This implies that anemia prevention and control strategies that target pregnant and lactating women could potentially have extended effect on reducing the burden of childhood anemia. Children of anemic mothers should be given especial attention in anemia intervention programs.

We found that anemia highly affected children of inferior wealth status. Consistent with our finding, many studies reported that children from poorest households were at higher risk of anemia [28–31, 68, 69]. A study that analyzed DHS data from 45 LMICs found that a higher prevalence of childhood anemia in the lower socioeconomic status in 37 of the 45 (82%) countries [69]. There are different possible explanations for the disproportionately higher prevalence of anemia among poor household. For example, Household with better wealth status consumes more iron rich food such as meat compared to lower income group [70]. The other reason could be related with the lower coverage for maternal, newborn, and child health interventions among the poorest quintiles [71]. In addition, although nutrition intervention policies improved nutritional quality in general, they failed to reduce socioeconomic inequalities with regard to nutrition [72, 73]. This indicates the need for designing inclusive anemia prevention and control strategy especially targeting the poor.

The current study should be interpreted in the context of the following strengths and limitations. Using nationally representative data and applying geospatial Bayesian statistics are strengths of this study. Accounting for spatial dependency when exploring risk factors of anemia is very important to improve the model fit for anemia model..DHS data lacks comprehensive information on the risk factors of anemia such as malaria, intestinal parasite and other important infection and inflammation biomarkers. The data also lacks adequate information to assess the dietary intake of important macro and micronutrient related with anemia. Furthermore, the dietary data were collected only from the last-born child. Hence, the lack of significant association between infection, diet and anemia should be interpreted with caution. In addition, because we relied solely on hemoglobin status to determine anemia, we could not distinguish whether the anemia was because of iron deficiency or other causes. Lastly, as this study was cross sectional survey, we could not disregard the possibility of reverse causality.

## Conclusions

In conclusion, malnutrition, maternal anemia, increased parity, and poor wealth status were risk factors of anemia among preschool children in Ethiopia. The existing anaemia control programs such as pregnancy IFA supplementation should be strengthened to halt intergenerational effect of anaemia. Furthermore, routine childhood anaemia screening and intervention program should be part of the Primary health care system in Ethiopia. Programs should target both mothers and children since we found strong association between maternal anemia and childhood anemia. Further researches are needed to understand etiologies of anemia and conduct intervention and implementation effectiveness trials for different settings of Ethiopia.

## Data Availability

The data that support the findings of this study are available from the DHS website at (https://dhsprogram.com).
